# Effect of Varicocele and Its Treatment on Testosterone in Hypogonadal Men with Varicocele: Review of the Literature

**DOI:** 10.4274/balkanmedj.galenos.2020.2020.1.85

**Published:** 2020-04-10

**Authors:** Selahittin Çayan, Erdem Akbay, Barış Saylam, Ateş Kadıoğlu

**Affiliations:** 1 Department of Urology, Mersin University School of Medicine, Mersin, Turkey; 2 Department of Urology, İstanbul University İstanbul School of Medicine, İstanbul, Turkey

**Keywords:** Adult, urinary tract, testicular, testosterone, varicocele

## Abstract

Varicocele might cause deterioration in Leydig cell functions, and it is a significant risk factor for hypogonadism. Some controversial issues have been raised in the treatment of hypogonadal men with varicoceles. Symptomatic hypogonadal men with varicoceles have two options: testosterone replacement therapy or varicocele treatment. Both approaches have some advantages and disadvantages. This review summarizes the effect of varicoceles on total plasma testosterone level and addresses whether varicocele repair is effective to improve testosterone levels in hypogonadal men with varicoceles. Experience from large clinical studies in the literature suggests that varicocele repair may increase serum testosterone level in men with varicoceles and testosterone deficiency. Varicocele repair could be offered to men with clinically palpable varicocele and hypogonadism. As the treatment method, microsurgical varicocele repair could be preferred to provide the best improvement. Another advantage of varicocele repair for hypogonadism, instead of exogenous testosterone treatment, is its ability to preserve the fertility status in men who may desire a child in the future. However, further studies are required to clarify varicocel-related Leydig cell dysfunction and to advise hypogonadal patients about the sufficient effectiveness of varicocele repair.

Varicocele might cause deterioration in Leydig cell functions, and it is a significant risk factor for hypogonadism. Some controversial issues have been raised in the treatment of hypogonadism and varicoceles. Symptomatic hypogonadal men with varicoceles have two options: testosterone replacement therapy or varicocele treatment. Both approaches have some advantages and disadvantages. Approximately 90% of men will remain azoospermic under exogenous testosterone treatment, leading to fertility issues. Despite discontinuation of exogenous testosterone treatment, 35% of symptomatic men might have irreversible azoospermia (1). In hypogonadal men who desire a child, stimulation of hypothalamic pituitary testis axis with gonadotropins and clomiphene citrate may be difficult to monitor serum hormone levels. For men who prefer varicocele repair, whether patients will have a sufficient increase in testosterone after varicocele repair remains unclear. Treatment success might depend on the treatment method (surgery or radiologic interventions) and complication rates, including varicocele recurrence and testicular atrophy.

Varicoceles can be treated in the presence of clinical findings such as inability to conceive, abnormality in semen parameters, and scrotal pain in case analgesics and anti-inflammatory drugs fail (2). Infertile men with hypogonadism and varicoceles can be managed with either treatment of varicocele via surgery (open, laparoscopic), radiologic embolization, or use of assisted reproductive technology (ART). ART, consisting of intrauterine insemination, in vitro fertilization, and intracytoplasmic sperm injection, is offered based on total motile sperm count. If varicocele treatment is preferred, improvement of testosterone level will take some time; if ART is preferred to conceive, testosterone treatment might be given to these hypogonadal men for late onset hypogonadism symptoms (3).

This review summarizes the efficacy of varicoceles on total plasma testosterone level and addresses whether treatment of varicocele is effective in the improvement of serum testosterone levels in men with hypogonadism and varicoceles.

## EVIDENCE FOR VARICOCELE-RELATED HYPOGONADISM AND EFFECT OF VARICOCELE TREATMENT ON TESTOSTERONE

Varicoceles might cause decreased testosterone synthesis and impaired sperm production, indicating a link between varicocele and hormonal dysfunction. Evidence for varicocele-related hypogonadism and effect of its treatment on testosterone have been demonstrated with experimental and clinical studies.

### Experimental studies

Experimental studies have shown impairment in Leydig cell functions in animal studies and decreased intratesticular testosterone in human testicular tissues and measurement of human testicular temperature in men with varicocele at the beginning and after the treatment of varicoceles.

Varicocele-related hyperthermia might cause impairment in the cholesterol synthesis pathway by defecting 17α- hydroxylase and 17,20-lyase enzyme inhibition, leading to increased reactive oxygen species level (4). As experimental evidence to show varicocele-related Leydig cell damage, Zheng et al. (5) divided 50 adolescent male rats into four groups, namely, varicocele, sham, artery ligating, and sparing varicocelectomy groups, to measure Johnen’s score, morphological structure of seminiferous tubules, and intratesticular testosterone level. They found that the presence of varicocele impairs Leydig cell function, and artery sparing varicocelectomy repairs Leydig cell function, but artery ligating varicocelectomy leads to additional deterioration.

In another rat study, Luo et al. (6) measured serum and intratesticular testosterone, as well as the apoptosis and expression of steroidogenetic acute regulatory (StAR) protein, which is a transporter of cholesterol and androgen products into the mitochondria. They found that varicoceles impair Leydig cell function by increasing apoptosis and suppressing the expression of the StAR protein.

In an experimental rat study, Rajfer et al. (7) compared measurements of intratesticular testosterone and υ4 pathways of testosterone synthesis, including 17-alpha-hydroxylase, 17,20- desmolase, and 17-βeta-hydroxysteroid dehydrogenase, between the time-dependent varicocele and control (sham) groups. They concluded that varicoceles exert detrimental effects on the biosynthesis of testosterone in both testes, and this effect occurs primarily at the 17,20-desmolase step.

Wright et al. (8) measured testicular temperature before and after microsurgical varicocele repair in 119 men with varicocele and 45 controls with no varicocele. They found that the presence of varicocele increases testicular temperatures on both sides even in men with unilateral and bilateral varicoceles. Microsurgical varicocele repair was observed to decrease testicular temperatures on both sides.

### Clinical studies

Several clinical studies have reported the effect of varicoceles on serum testosterone. Ando et al. (4) compared measurements of serum gonadotropins, testosterone, 17-OH-progesterone, dihydrotestosterone, and estradiol, as well as the hormonal response to human chorionic gonadotropin stimulation between 108 patients with varicocele and 46 controls. They found significantly increased 17-hydroxy-progesterone and significantly decreased testosterone in patients with varicoceles compared with the controls. They concluded that the presence of varicoceles affects testicular hormone production due to enzymatic impairment.

In the literature, very limited clinical studies have focused on the effects of varicocele repair on serum testosterone and effects of varicocele repair on sexual functions in hypogonadal men with varicoceles. The first study in 1975 reported that 10 out of 33 (30%) men with varicoceles had decreased testosterone level and erectile dysfunction, and both symptoms improved in those men after varicocelectomy (9). Rodriguez-Rigau et al. (10) in 1978 reported that men with varicoceles and normal range of testosterone level demonstrated decreased Leydig cell counts on testicular biopsy in men who underwent varicocelectomy. In addition to total plasma testosterone level, we demonstrated that men with varicoceles exhibited decreased free testosterone level and increased plasma follicle stimulating hormone (FSH) level; after microsurgical varicocele repair, total plasma and free testosterone levels significantly increased, and FSH level significantly decreased (11). In 2011, Tanrikut et al. (12) demonstrated that men with varicoceles have lower serum total testosterone level compared with controls. Of the men with low total serum testosterone level, 79% had normal total serum testosterone level after varicocelectomy, and 16% of controls had normal testosterone level over the same period.

Hayden and Tanrikut (13) reviewed 18 studies to investigate the effect of varicocele repair on total serum testosterone level. Although symptomatic hypogonadal men showed greater improvement in postoperative serum testosterone levels, the presence of low testosterone level in men with varicoceles remains a controversial indication for repairing varicoceles.

Li et al. (14) reported a meta-analysis consisting of nine studies with 814 patients to investigate the effect of varicocele treatment on testosterone level in infertile men with varicoceles. They found that the mean total plasma testosterone level increased to 97.48 ng/dL after varicocele repair, suggesting an improvement in Leydig cell functions.

Guercio et al. (15) investigated all indications for varicocele repair in a contemporary cohort of 18-45-year-old men with varicocele by using a commercial database in the USA. They found semen analysis [odds ratio (OR): 2.78] and serum testosterone evaluation (OR: 1.67) as the strongest predictors of varicocele repair. Therefore, although presence of fertility problems remains the number one indication for varicocele repair, the presence of decreased testosterone level is an independent predictor in the clinical practice to manage varicoceles in the USA.

Hsiao et al. (16) included 78 patients with varicoceles who underwent microsurgical varicocele repair and compared serum testosterone level from pre-treatment to post-treatment on the basis of varicocele grades. They found that 83% of the patients exhibited an increase in postoperative testosterone, and varicocele repair provided increased level of serum testosterone, regardless of the varicocele grading. Repair of even small varicoceles provided increased serum testosterone levels. In another study, the same group compared serum testosterone level according to patients’ age groups in 272 patients with varicocele and testosterone level of <400 ng/dL who underwent microsurgical varicocele repair (17). They found that surgical treatment of varicoceles significantly improved sperm parameters and testosterone even in elderly men; therefore, varicocele repair could be offered to older infertile men with hypogonadism (17).

Zohdy et al. (18) reported 141 infertile men with varicoceles and compared total serum testosterone and international index of erectile function (IIEF-5) score from the beginning to the end of the study, which involved 103 men who underwent sub-inguinal microsurgical varicocelectomy and 38 men who preferred ART. They found that the IIEF-5 scores significantly increased from 17.1±2.6 to 19.7±1.8, and serum testosterone significantly increased from 379.1±205.8 ng/dL to 450.1±170.2 ng/dL; 75.5% of the patients presented normal testosterone levels following varicocele repair. However, no significant change was found in the patients with varicocele who preferred ART.

Tanrikut et al. (12) included 325 men with varicoceles and 510 controls (vasectomy reversal) to compare total serum testosterone by age before and after varicocele repair. They found that plasma testosterone level significantly increased from 358±126 ng/dL to 454±168 ng/dL after surgery, and 70% had increased level of testosterone (increase of 0%-50% in 41%, 51%-100% in 19%, and >100% in 10%). Therefore, varicoceles might cause androgen deficiency, and treatment of varicoceles might increase testosterone levels in hypogonadal men with varicoceles.

Abdel-Meguid et al. (19) conducted a prospective controlled study with four groups to compare total serum testosterone at 6 and 12 months: infertile men with and without varicoceles (hypogonadal/ eugonadal) and fertile men with and without varicoceles (hypogonadal/eugonadal). They found significantly lower testosterone level in men with varicoceles compared with normal men. Varicocelectomy significantly increased total testosterone among hypogonadal men but showed no improvement in eugonadal men. Increased testosterone levels showed a strong correlation with preoperative testosterone and sperm concentrations.

Total serum testosterone can even increase after treating recurrent varicoceles. Çayan and Akbay (20) divided 217 infertile men with recurrent varicoceles into two groups: 120 underwent redo microsurgical sub-inguinal varicocele repair, and 97 were observed only as the control group. In their study, postoperative semen parameters, total serum testosterone level, and spontaneous pregnancy rates significantly improved with microsurgical varicocele repair for the recurrence of varicocele compared with the controls. Pregnancy was achieved in 52.5% of the couples in the surgical treatment group and in 39.2% of the control group. The surgical group had a mean increase of 1.36 ng/mL in total testosterone, whereas the control group showed a mean decrease of 0.23 ng/mL. Treatment of recurrent varicoceles also decreased the need for use and level of ART (20).

In one meta-analysis, Chen et al. (21) included seven studies, consisting of 712 patients, to compare pre-and post-surgical serum testosterone levels. The mean postoperative serum testosterone improved by 34.3 ng/dL compared with pre-treatment levels. The mean testosterone level increased by 105.65 ng/dL in the hypogonadal men, favoring varicocele repair (21). However, results must be treated with caution, and adequate cost benefit analysis must be undertaken to determine the risks and benefits of surgical intervention over exogenous testosterone treatment in this setting.

Table 1 shows all studies in the literature, reporting the effect of microsurgical varicocele repair on serum testosterone level in hypogonadal men with clinical varicocele. All of the six studies included in the table provided significant improvement in the mean total serum testosterone level after microsurgical varicocele repair in hypogonadal men with varicocele (12,16,17,18,19,22).

## NOVEL TREATMENT OPTIONS FOR VARICOCELES IN HYPOGONADAL MEN

Hypogonadal men with varicoceles can be treated with open surgery, radiologic interventions, and laparoscopic approaches (23,24). When infertile men with varicoceles are treated, the best seminal improvement is aimed with the lowest complication rates such as varicocele recurrence and hydrocele and testicular atrophy. Studies suggested that only 35%-50% of men who undergo varicocele treatment exhibit a positive seminal response to varicocele treatment, and others show a negative response to varicocele treatment due to varicocele recurrence, genetic disorders, and technical failure (23,24). Table 2 shows complications and fertility outcomes of the studies in the literature for the treatment of varicoceles.

Advantages of the radiologic interventions for the treatment of varicoceles are rapid recovery period and relatively low cost, but such interventions include interventional failure to enter the access sheath, contrast agent allergy, thrombosis, and high recurrence rate due to anatomical vein structures. The laparoscopic approach could provide a faster postoperative recovery period than radiologic interventions. However, the disadvantages of laparoscopic and Palomo techniques include no possibility to ligate external spermatic veins that may cause persistent or recurrence of varicoceles. In addition to ligation of the internal spermatic vein branches, open surgical inguinal and sub-inguinal approaches facilitate the ligation of the external spermatic vein. The sub-inguinal approach provides less postoperative pain than the inguinal approach, because the aponeurosis of the external oblique muscle is not opened with the sub-inguinal approach. However, the inguinal approach ligates a lower number of internal spermatic vein channels than the sub-inguinal approach. Moreover, the arteries can be preserved easily at the proximal level, because of the low number of artery branches compared with the distal level with the sub-inguinal approach.

All treatment methods for primary varicoceles were compared in a previous meta-analysis (23). This meta-analysis demonstrated that the highest spontaneous pregnancy rate was achieved with the microsurgical varicocelectomy series (42%). The spontaneous pregnancy rates were in the following order: 37.7% for the Palomo technique series, 36% for the macroscopic inguinal varicocelectomy series, 33.2% for radiologic interventions, and 30% for laparoscopic varicocelectomy techniques. This meta-analysis also demonstrated the lowest complication rates with the microsurgical varicocelectomy techniques (1.05% for recurrence of varicocele and 0.44% for hydrocele formation) compared with other varicocelectomy methods.

Varicocele might cause deterioration in Leydig cell functions, and it is a significant risk factor for hypogonadism. Experience from large clinical studies in the literature suggests that varicocele repair may increase serum testosterone level in men with varicoceles and testosterone deficiency. Approximately 60%-80% of men with low serum testosterone will exhibit normalization of testosterone level after varicocele repair. Varicocele repair could be offered to men with clinically palpable varicocele and hypogonadism. As the treatment method, microsurgical varicocele repair might provide the most improvement in total serum testosterone level compared with other varicocelectomy methods.

Another advantage of varicocele repair for hypogonadism, instead of exogenous testosterone treatment, would be saving the fertility status in men who may desire a child in the future. However, further prospective randomized controlled studies are required to clarify varicocele-related Leydig cell dysfunction and advise hypogonadal patients about the sufficient effectiveness of varicocele repair.

## Figures and Tables

**Table 1:**
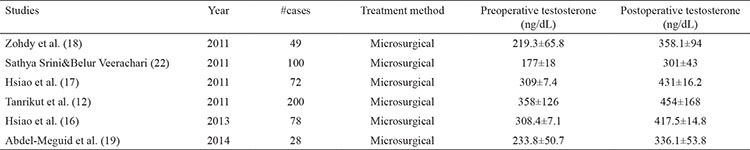
Studies in the literature reporting the effect of varicocele repair on serum testosterone level in hypogonadal men with clinical varicocele

**Table 2:**
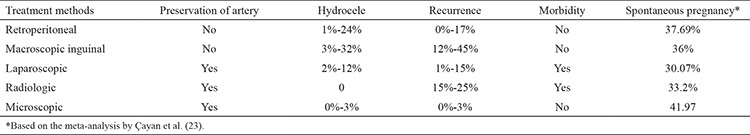
Complications and fertility outcomes of the studies in the literature for the treatment of varicocele

## References

[1] Samplaski MK, Loai Y, Wong K, Lo KC, Grober ED, Jarvi KA (2014). Testosterone use in the male infertility population: prescribing patterns and effects on semen and hormonal parameters. Fertil Steril.

[2] Schlegel PN, Goldstein M (2011). Alternate indications for varicocele repair: non-obstructive azoospermia, pain, androgen deficiency and progressive testicular dysfunction. Fertil Steril.

[3] Practice Committee of the American Society for Reproductive Medicine (201). Management of nonobstructive azoospermia: a committee opinion. Fertil Steril.

[4] Ando S, Giacchetto C, Colpi G, Beraldi E, Panno ML, Lombardi A, Sposato G (1984). Physiopathologic aspects of Leydig cell function in varicocele patients. J Androl.

[5] Zheng YQ, Zhang XB, Zhou JQ, Cheng F, Rao T, Yao Y (2008). The effects of artery-ligating and artery-preserving varicocelectomy on the ipsilateral testes in rats. Urology.

[6] Luo DY, Yang G, Liu JJ, Yang YR, Dong Q (2011). Effects of varicocele on testosterone, apoptosis and expression of StAR mRNA in rat Leydig cells. Asian J Androl.

[7] Rajfer J, Turner TT, Rivera F, Howards SS, Sikka S (1987). Inhibition of testicular testosterone biosynthesis following experimental varicocele in rats. Biol Reprod.

[8] Wright EJ, Young GPH, Goldstein M (1997). Reduction in testicular temperature after varicocelectomy in infertile men. Urology.

[9] Comhaire F, Vermeulen A (1975). Plasma testosterone in patients with varicocele and sexual inadequacy. J Clin Endocrinol Metab.

[10] Rodriguez-Rigau LJ, Weiss DB, Zukerman Z, Grotjan HE, Smith KD, Steinberger E (1978). A possible mechanism for the detrimental effect of varicocele on testicular function in man. Fertil Steril.

[11] Çayan S, Kadioglu A, Orhan I, Kandirali E, Tefekli A, Tellaloglu S (1999). The effect of microsurgical varicocelectomy on serum follicle stimulating hormone, testosterone and free testosterone levels in infertile men with varicocele. BJU Int.

[12] Tanrikut C, Goldstein M, Rosoff JS, Lee RK, Nelson CJ, Mulhall JP (2011). Varicocele as a risk factor for androgen deficiency and effect of repair. BJU Int.

[13] Hayden RP, Tanrikut C (2016). Testosterone and varicocele. Urol Clin N Am.

[14] Li F, Yue H, Yamaguchi K, Okada K, Matsushita K, Ando M, Chiba K, Fujisawa M (2012). Effect of surgical repair on testosterone production in infertile men with varicocele: a meta-analysis. Int J Urol.

[15] Guercio C, Patil D, Mehta A (2019). Hypogonadism is independently associated with varicocele repair in a contemporary cohort of men in the USA. Asian J Androl.

[16] Hsiao W, Rosoff JS, Pale JR, Powell JL, Goldstein M (2013). Varicocelectomy is associated with increases in serum testosterone independent of clinical grade. Urology.

[17] Hsiao W, Rosoff JS, Pale JR, Greenwood EA, Goldstein M (2011). Older age is associated with similar improvements in semen parameters and testosterone after subinguinal microsurgical varicocelectomy. J Urol.

[18] Zohdy W, Ghazi S, Arafa M (2011). Impact of varicocelectomy on gonadal and erectile functions in men with hypogonadism and infertility. J Sex Med.

[19] Abdel-Meguid TA, Farsi HM, Al-Sayyad A, Tayib A, Mosli HA, Halawani AH (2014). Effects of varicocele on serum testosterone and changes of testosterone after varicocelectomy: a prospective controlled study. Urology.

[20] Çayan S, Akbay E (2018). Fate of recurrent or persistent varicocele in the era of assisted reproduction technology: Microsurgical subinguinal redo varicocelectomy versus observation. Urology.

[21] Chen X, Yang D, Lin G, Bao J, Wang J, Tan W (2017). Efficacy of varicocelectomy in the treatment of hypogonadism in subfertile males with clinical varicocele: A metaanalysis. Andrologia.

[22] Sathya Srini V, Belur Veerachari S (2011). Does varicocelectomy improve gonadal function in men with hypogonadism and infertility? Analysis of a prospective study. Int J Endocrinol.

[23] Çayan S, Shavakhabov S, Kadioglu A (2009). Treatment of palpable varicocele in infertile men: a meta-analysis to define best technique. J Androl.

[24] Rotker K, Sigman M (2016). Recurrent varicocele. Asian J Androl.

